# Constitutive phosphorylation of the mTORC2/Akt/4E-BP1 pathway in newly derived canine hemangiosarcoma cell lines

**DOI:** 10.1186/1746-6148-8-128

**Published:** 2012-07-29

**Authors:** Atsuko Murai, Samah Abou Asa, Atsushi Kodama, Akihiro Hirata, Tokuma Yanai, Hiroki Sakai

**Affiliations:** 1 Laboratory of Veterinary Pathology, Department of Veterinary Medicine, Faculty of Applied Biological Sciences, Gifu University, 1-1 Yanagido, Gifu, 501-1193, Japan; 2 Research Fellow of the Japan Society for the Promotion of Science, 1-8 Chiyoda, Tokyo, 102-8472, Japan; 3 Comparative Cancer Center, Faculty of Applied Biological Sciences, Gifu University, 1-1 Yanagido, Gifu, 501-1193, Japan

**Keywords:** Cell line, Canine, Akt, Hemangiosarcoma, mTOR, 4E-BP1

## Abstract

**Background:**

Canine hemangiosarcoma (HSA) is a malignant tumor with poor long-term prognosis due to development of metastasis despite aggressive treatment. The phosphatidyl-inositol-3 kinase/Akt/mammalian target of rapamycin (PI3K/Akt/mTOR) pathway is involved in its endothelial pathologies; however, it remains unknown how this pathway plays a role in canine HSA. Here, we characterized new canine HSA cell lines derived from nude mice-xenografted canine HSAs and investigated the deregulation of the signaling pathways in these cell lines.

**Results:**

Seven canine HSA cell lines were established from 3 xenograft canine HSAs and showed characteristics of endothelial cells (ECs), that is, uptake of acetylated low-density lipoprotein and expression of canine-specific CD31 mRNA. They showed varied morphologies and mRNA expression levels for VEGF-A, bFGF, HGF, IGF-I, EGF, PDGF-B, and their receptors. Cell proliferation was stimulated by these growth factors and fetal bovine serum (FBS) in 1 cell line and by FBS alone in 3 cell lines. However, cell proliferation was not stimulated by growth factors and FBS in the remaining 3 cell lines. Phosphorylated p44/42 Erk1/2 was increased by FBS stimulation in 4 cell lines. In contrast, phosphorylation of Akt at Ser^473^, mTOR complex 1 (mTORC1) at Ser^2448^, and eukaryotic translation initiation factor 4E-binding protein 1 (4E-BP1) at Ser^65^ was high in serum-starved condition and not altered by FBS stimulation in 6 cell lines, despite increased phosphorylation of these residues in normal canine ECs. This suggested that the mTORC2/Akt/4E-BP1 pathway was constitutively activated in these 6 canine HSA cell lines. After cell inoculation into nude mice, canine HSA tumors were formed from 4 cell lines and showed Akt and 4E-BP1 phosphorylation identical to the parental cell lines.

**Conclusions:**

Our findings suggest that the present cell lines may be useful tools for investigating the role of the mTORC2/Akt/4E-BP1 pathway in canine HSA formation both *in vivo* and *in vitro*.

## Background

Hemangiosarcoma (HSA) is a malignant tumor derived from endothelial cells (ECs). Canine HSAs easily metastasize to other organs, and the mean survival time is less than 6 months even with surgical and chemotherapeutic interventions [1]. Human angiosarcomas are also aggressive tumors that show a propensity for distant metastasis [2]. Angiosarcomas occur rarely in humans, and no effective treatments have yet been developed. Because HSAs occur more commonly in dogs than in humans [1], it may be easier to study the progression of these tumors in dogs and to establish effective treatments that may also be applicable for human angiosarcomas.

Vascular endothelial growth factor (VEGF) and basic fibroblast growth factor (bFGF), along with their receptors, are overexpressed in human angiosarcomas and canine HSAs [2,3]. These growth factors usually activate receptor tyrosine kinases (RTKs), which in turn activate downstream signaling pathways. Among these signaling pathways, MAPK/Erk and phosphatidyl-inositol-3 kinase /Akt/mammalian target of rapamycin (PI3K/Akt/mTOR) are the major oncogenic signaling pathways [4,5]. The MAPK/Erk pathway has been reported to be highly upregulated in benign endothelial tumors rather than in malignant tumors [6,7]. In contrast, the PI3K/Akt pathway is known to be one of the important pathways in the manifestation of endothelial pathologies. For example, activated or mutated PI3K/Akt causes the development of HSA in chickens [8]. Mutation of *PTEN*, a PI3K antagonist, has been reported in canine HSAs [9] and human angiosarcomas [10]. Moreover, the Akt/mTOR pathway is upregulated in sporadic angiosarcomas in humans [11]. However, the role of the PI3K/Akt/mTOR pathway has not been investigated in canine HSAs.

mTOR, a serine/threonine kinase, is highly conserved among animal species and regulates cell growth and cell cycle progression by controlling cap-dependent translation [12,13]. mTOR exists as 2 distinct multi-protein complexes, mTOR complex 1 (mTORC1) and mTORC2. mTORC1, consisting of mTOR, raptor, and mLST8 (also known as GβL), is located downstream of PI3K/Akt and is activated by Akt via phophorylation at Ser^2448^[14]. mTORC1 in turn phosphorylates the eukaryotic translation initiation factor 4E (eIF4E)-binding protein 1 (4E-BP1) and S6 kinase (S6K) [13]. In its hypophosphorylated state, 4E-BP1 binds to and inhibits the activity of eIF4E, and 4E-BP1 phosphorylation induces the release of 4E-BP1 from eIF4E, which leads to subsequent mRNA translation [15]. eIF4E is known to selectively stimulate several malignancy-related transcripts, including cyclin D1, bFGF, and VEGF [16], which are involved in growth, survival, and angiogenesis and are known to be overexpressed in human angiosarcomas [2,17] and canine HSAs [3,18]. mTORC2, consisting of mTOR, rictor, and mLST8, is located upstream of Akt and phosphorylates Akt at Ser^473^[19]. Although RTK signaling is known to activate mTORC2 through the PI3K/PTEN pathway, less is known about mTORC2 signaling compared with that for mTORC1 [12,20].

Because of the limited availability of human angiosarcoma [21,22] or canine HSA [23,24] cell lines, it was difficult to study deregulated signaling pathways in these tumors. We recently established xenograft canine HSA tumors from nude mice [25] and, in the present study, we present 7 canine HSA cell lines derived from the xenograft tumors. By using these established cell lines, we characterized the biological behavior of the cells in response to growth factors and disruption of signaling pathways. The primary aim of these studies is the identification of novel molecular targets for the treatment of canine HSAs.

## Methods

### Cell culture

To establish canine HSA cell lines, we used 3 xenograft canine HSA tumors, which were established from 3 spontaneous canine HSAs as described previously [25]. Briefly, the xenograft tumor Ju was established from HSA tissue in the liver of a 10-year-old Labrador Retriever, Re was established from HSA tissue in the right atrium of a 10-year-old Golden Retriever, and Ud was established from HSA tissue in the spleen of an 11-year-old Papillion. These tumor tissues were subcutaneously transplanted into the right and left dorsal area of the trunk of 3-week-old male KSN/Slc nude mice (Japan SLC, Inc., Hamamatsu, Japan), and xenograft models were established after >5 passages. The xenografted tumor tissues were minced and sequentially digested in 0.1% collagenase Type I (Gibco, CA, USA) at 37 °C for 15 min, and then 0.25% trypsin-EDTA (Gibco) at 37 °C for 15 min. The cell suspension was subsequently filtered through a 70-μm cell strainer (BD Biosciences, NJ, USA), and then resuspended in Medium 199 (Gibco) supplemented with 10% fetal bovine serum (FBS, AusGeneX, Oxenford, Australia). The cells were cultured in a humidified incubator at 37 °C with 5% CO_2_. Subconfluent cells were passaged after detachment with 0.25% trypsin-EDTA, and cell lines were established after >60 passages. For cloning, one cell per well was plated in separate 96-well plates (Thermo Scientific, MA, USA).

For measuring the growth curve and population doublings, the established cell lines were plated in 24-well plates (Thermo Scientific) at 5000 cells/well in 1 mL of Medium 199 containing 10% FBS. The cells were trypsinized and counted with a hemocytometer using trypan blue every 24 h. Triplicate wells were used for counting each cell line.

To examine the uptake of the acetylated low density lipoprotein (Ac-LDL) in HSA cell lines, subconfluent cells were incubated with 10 μg/mL DiI-Ac-LDL (Biomedical Technologies Inc., MA, USA) at 37 °C for 4 h in Medium 199 according to the manufacturer’s instructions. After washing, the cells were observed with an inverted fluorescent microscope (Biozero BZ-8000, KEYENCE, Osaka, Japan) with a rhodamine filter. Human umbilical vein endothelial cells (HUVECs, Cell Applications Inc., CA, USA) were purchased and used as a positive control.

### ELISA

For measuring growth factors in cell supernatant, HSA cell lines were cultured under standard conditions in Medium 199 containing 10% FBS. After incubation for 72 h, the plates were washed with Hanks’ Balanced Salt Solution (HBSS, Sigma-Aldrich, MO, USA), and the medium was changed to Medium 199 containing 1% FBS. After further incubation for 24 h, the supernatant was stored at −80 °C. The cells were trypsinized and counted with a hemocytometer using trypan blue. VEGF-A and bFGF concentrations in cell supernatant were determined using commercial ELISA kits for humans (Quantikine, R&D Systems, MN, USA) according to the manufacturer’s instructions since these kits were previously shown to have cross-reactivity with canine growth factors [26,27].

### Immunocytochemistry

Canine HSA cell lines were cultured to subconfluence under standard conditions in Medium 199 containing 10% FBS and were used for protein expression for VEGF-A and bFGF. After washing with phosphate-buffered saline without Ca^2+^ or Mg^2+^ [PBS (−)], the cells were incubated with Protein Block Serum-Free (Dako, Kyoto, Japan) for 30 min at room temperature (RT). The cells were incubated overnight at 4 °C with primary antibodies for VEGF-A (mouse monoclonal antibody clone C-1, 1:50; Santa Cruz Biotechnology Inc., Santa Cruz, CA, USA) and bFGF (rabbit polyclonal antibody, 1:200; Santa Cruz Biotechnology Inc.). The specific protein signals were visualized using the 3,3′-diaminobenzidinetetrahydrochloride (Liquid DAB + Substrate Chromogen System, Dako). The cells were counter-stained with Mayer’s hematoxylin.

### Reverse transcriptase-polymerase chain reaction (RT-PCR)

Expression of mRNA for growth factors and their receptors was examined in the established cell lines. Total RNA was extracted from subconfluent cells grown in Medium 199 containing 10% FBS using TRIzol reagent (Gibco). Reverse transcriptase-polymerase chain reaction was performed as previously described [25] using the OneStep RT-PCR kit (Qiagen, Hilden, Germany). RT-PCR was carried out in a Thermal Cycler Dice Gradient (Takara, Ohtsu, Japan). Amplifications were performed under the following conditions: reverse-transcription reaction for 30 min at 50 °C, an initial polymerase activation step for 15 min at 95 °C, denaturation for 30 s at 95 °C, annealing for 30 s, and extension for 1 min at 72 °C. To confirm the absence of genomic DNA contamination, RT-PCR was carried out for DNase I-treated total RNA with One Step Enzyme Mix that had been deactivated for reverse transcription activity by heating for 15 min at 95 °C. The primer sequences, annealing temperatures, annealing cycle number, and product sizes used are listed in Table 1. The primers were generated from canine-specific sequences as previously described [25].

**Table 1:** PCR primers and conditions

**Gene**	**Primer sequence**	**Annealing temperature (°C)**	**product size (bp)**
CD31	For: 5'-GCACACAAGAGGCATGGTAAC-3'	63.0	211
	Rev: 5'-GAATGGAGCACCACAGGTTT-3'		
vWF	For: 5'-GCAATGTCTCCTCTGATGAAG-3'	63.0	221
	Rev: 5'-GTACAAGACAACCCCCTGCT-3'		
VEGF-A	For: 5'-CGTGCCCACTGAGGAGTT-3'	64.0	249, 231, and 177
	Rev: 5'-AAATGCTTTCTCCGCTCTGA-3'		
flt-1	For: 5'-ACCCTAAAGAAAGGCCAAGA-3'	63.0	156
	Rev: 5'-CATCAGAGAAGGCAGGAGATG-3'		
flk-1	For: 5'-GGAGCTCCAGAATGTGTCCT-3'	66.0	187
	Rev: 5'-GGTGCATGAAACTTCAATGGT-3'		
bFGF	For: 5'-CACTTCAAGGACCCCAAGAG-3'	61.0	234
	Rev: 5'-GAAGCACTCGTCAGTAACACAT-3'		
FGFR-1	For: 5'-GAAGTCGGATGCTACAGAGAAA-3'	65.0	162
	Rev: 5'-CGTAAGTTGCCTTTGGAAGC-3'		
HGF	For: 5'-ATGGGGAATGAGAAATGCAG-3′	60.0	210
	Rev: 5'-AAAAATGCCAGGACGATTTG-3′		
c-Met	For: 5-GATCTGGGCAGTGAATTAGT-3′	58.0	417
	Rev: 5-GTCCAACAAAGTCCCATGAT-3′		
IGF-I	For: 5'-AAGCAGCACTCATCCACGAT-3'	64.0	281
	Rev: 5'-CAGCAGTCTTCCAACCCAAT-3'		
IGF-IR	For: 5'-ACAACTACGCCCTGGTCATC-3'	64.0	295
	Rev: 5'-CAGCGATTTGTAGTCCAGCA-3'		
EGF	For: 5'-CTGTGGGATGCAGTACATGG-3'	61.0	204
	Rev: 5'-CTCGGTAGCCTTCTGAGCAC-3'		
EGF-R	For: 5′-AGGAGAGGAGAACTGCCAGA-3′	63.0	250
	Rev: 5′-CAGGTGGCACCAAAGCTGTA-3′		
PDGFB	For: 5'-TTGTACGGAAGAAGCCAACC-3'	64.0	279
	Rev: 5'-CCTCAATCTCCTCCAGATGC-3'		
PDGFR-α	For: 5'-GCCCCATTTACATCATCACC-3'	64.0	213
	Rev: 5'-TGTCAGCTTGCTTCATGTCC-3'		
PDGFR-β	For: 5'-ATGCAGTGCAGACTGTGGTC-3'	59.0	190
	Rev: 5'-TCAGCACTAGGGATGTGCAG-3'		
β-actin	For: 5'-ATTGAGCACGGCATCGTC-3'	65.5	261
	Rev: 5'-GTCACCGGAGTCCATCACG-3'		

### Cell proliferation assays

Cell proliferation assays were performed as previously described [24]. Briefly, the established cell lines were plated at 1 × 10^3^ cells per well in 200 μL Medium 199 containing 10% FBS in 96-well plates for 24 h. The cells were washed with HBSS, and the medium was replaced with Medium 199 containing 1% FBS. After 24 h of serum starvation, the cells were mixed with 0, 1, 10, 50, or 100 ng/mL of growth factor in Medium 199 containing 1% FBS or were changed to Medium 199 containing 10% FBS. Growth factors included recombinant human VEGF, bFGF, IGF-I, HGF, EGF, or PDGF-BB (R&D Systems), and all of these have been reported to induce cell growth in canine HSA cell lines except VEGF and PDGF-BB [24]. Recombinant canine VEGF and HGF (R&D Systems) were also used. After 72-h incubation with growth factor or FBS, the relative viable cell number was assessed with the WST-1 assay (Roche Diagnostics, Mannheim, Germany) according to the manufacturer’s instructions. Each experiment was repeated three times. Canine aortic endothelial cells (CnAOECs, Cell Applications Inc.) were purchased and used to examine the cell growth of normal canine ECs.

### Western blotting

Canine HSA cell lines were cultured to 70–80% confluence under standard conditions in Medium 199 containing 10% FBS. Cells were then washed with HBSS and the medium was replaced with Medium 199 containing 1% FBS. After serum starvation for 24 h, the medium was replaced with Medium 199 containing 1% FBS or Medium 199 containing 10% FBS for 30 min. For PTEN expression, subconfluent cells grown in Medium 199 containing 10% FBS were used. After washing with PBS (−), the cells were lysed with RIPA Lysis Buffer (Santa Cruz Biotechnology Inc.) with Phosphatase Inhibitor Cocktail 2 and 3 (Sigma-Aldrich). The concentrations of whole cell lysates were determined by modified Lowry method using the DC protein assay kit (Bio Rad, CA, USA). Equal amounts of protein (10 μg) were subjected to sodium dodecyl sulphate-polyacrylamide gel electrophoresis (SDS-PAGE) under reducing conditions on 10% polyacrylamide gels. After separation by SDS-PAGE, the proteins were transferred onto a PVFD-membrane (Millipore, MA, USA). Membranes were blocked with 2% ECL Blocking Agent (GE Healthcare Life Sciences, Buckinghamshire, UK) in Tris-buffered saline containing 0.1% Tween 20 (TBS-T) for 1 h at RT. The membranes were then incubated overnight at 4 °C with primary antibodies for phosphorylated Akt (p-Akt Ser^473^, rabbit monoclonal antibody clone D9E, 1:1000, and Thr^308^, rabbit polyclonal antibody, 1:500; Cell Signaling Technology, MA, USA), Akt (rabbit polyclonal antibody, 1:1000; Cell Signaling Technology), p-p44/42 Erk1/2 (Thr^202^/Tyr^204^, rabbit polyclonal antibody, 1:1000; Cell Signaling Technology), p44/42 Erk1/2 (rabbit monoclonal antibody clone 137F5, 1:1000; Cell Signaling Technology), p-mTOR (Ser^2448^, rabbit monoclonal antibody clone D9C2, 1:1000; Cell Signaling Technology), mTOR (rabbit monoclonal antibody clone 7C10, 1:1000; Cell Signaling Technology), p-p70S6K (Thr^389^, mouse monoclonal antibody clone 1A5, 1:1000; Cell Signaling Technology), p70S6K (rabbit monoclonal antibody clone 49D7, 1:1000; Cell Signaling Technology), p-4E-BP1 (Thr^37/46^, rabbit monoclonal antibody clone 236B4, Thr^70^, rabbit polyclonal antibody, and Ser^65^, rabbit polyclonal antibody, all 1:1000; Cell Signaling Technology), 4E-BP1 (rabbit monoclonal antibody clone 53H11, 1:4000; Cell Signaling Technology), and PTEN (mouse monoclonal antibody clone A2B1, 1:200; Santa Cruz Biotechnology, Inc.). β-actin (mouse monoclonal antibody clone AC-15, 1:3000; Sigma-Aldrich) was used as a loading control. The specific protein signals were visualized with horseradish peroxidase-conjugated secondary antibodies using the ECL Plus Western Blotting Detection System (GE Healthcare). CnAOECs were used to examine the protein expression for normal canine ECs.

### Inoculation of cells and immunohistochemical staining

The established cell lines were harvested during logarithmic growth and prepared for injection in mice. Before injection, cells were trypsinized, counted, and washed twice with sterile PBS (−). A total of 1 × 10^6^ cells were suspended in 0.2 ml of PBS (−) and injected subcutaneously into the right and left dorsal area of the trunk of 3-week-old male KSN/Slc mice. Five mice were used for each cell line. The mice were observed for tumor development twice a week, and the size of the resulting tumor was measured. After 9 weeks, or when the tumors grew to 10 mm in diameter, the mice were humanely sacrificed, and the tumors were immediately removed. If a detectable tumor was not formed in the mice within 30 days, the mice were sacrificed at this time. The removed tumors were fixed in 10% neutral buffered formalin, embedded in paraffin, sectioned, and stained with hematoxylin and eosin (HE) or used for immunohistochemical staining. Immunohistochemical staining was performed for CD31 (prediluted mouse monoclonal antibody clone JC70A; Dako), von Willebrand factor (vWF, prediluted rabbit polyclonal antibody; Dako), Ki-67 antigen (mouse monoclonal antibody clone MIB-1, 1:25; Dako), p-Akt (Ser^473^, rabbit polyclonal antibody, rabbit monoclonal antibody clone D9E, 1:50, Thr^308^, rabbit polyclonal antibody, 1:50; Cell Signaling Technology), and p-4E-BP1 (Thr^37/46^, rabbit monoclonal antibody clone 236B4, 1:1600; Cell Signaling Technology) on all tumors formed from the cell injections. The experiments were performed according to the guidelines for the care and use of laboratory animals and approved by the Committee for Animal Research and Welfare of Gifu University (No. 08015).

### Statistical analysis

Student’s *t* test was used to determine statistical significance of the differences between the control and experimental data for the cell proliferation assay. Differences were considered statistically significant at p value of <0.05.

## Results

### Morphology and growth of canine HSA cell lines

**Figure 1:**
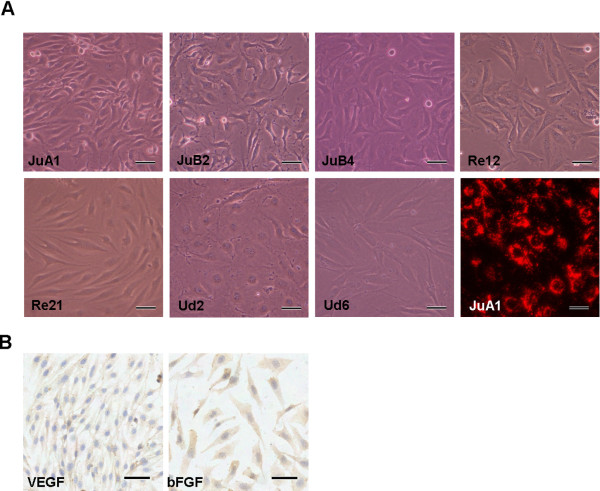
**Morphology, DiI-Ac-LDL uptake, and immunocytochemical staining for VEGF-A and bFGF of canine HSA cell lines.** (**A**) Morphological appearance and uptake of DiI-Ac-LDL (right bottom) of established canine HSA cell lines. Canine HSA cell lines were grown in Medium 199 with 10% FBS. Each cell line exhibited a different morphology. Bars = 50 μm. Right bottom; Uptake of DiI-Ac-LDL in KDM/JuA1. Bar = 25 μm. (**B**) Immunocytochemical staining for VEGF-A and bFGF. The cytoplasm of cells showing positive staining with VEGF-A in KDM/JuA1 and bFGF in KDM/Re12. Bars = 50 μm.

After 60 passages, 3 cell lines were established from the 3 xenograft tumors (Ju, Re, and Ud). After cloning, 7 sub-lines with differential morphologies were established from these 3 initial cell lines (Figure 1A). Three of the sub-lines, KDM/JuA1, KDM/JuB2, and KDM/JuB4, were established from a xenograft tumor of Ju, and the cells had spindle to polygonal cytoplasm with round to oval nuclei. Two sub-lines were established from a xenograft tumor of Re; KDM/Re12 cells had uniform stellate cytoplasm with oval nuclei, and KDM/Re21 cells had spindle cytoplasm with oval nuclei. Two sub-lines were established from a xenograft tumor of Ud; KDM/Ud2 cells had large polygonal cytoplasm with round nuclei, and KDM/Ud6 cells had spindle to polygonal cytoplasm with oval nuclei. All sub-lines took up DiI-Ac-LDL, which is used for identification of both normal and neoplastic ECs [21,22,28] (Figure 1A).

**Figure 2:**
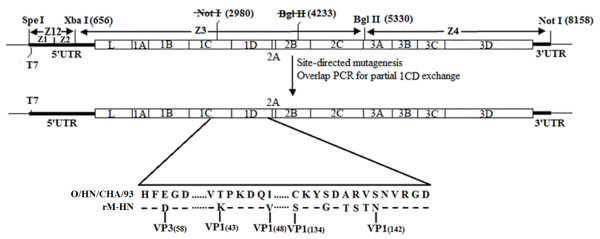
**Cell growth under standard conditions.** Growth curves of canine HSA cell lines. Each cell line was plated at 5000 cells/well in 1 mL of Medium 199 containing 10% FBS. The cells were trypsinized and counted with a hemocytometer using trypan blue every 24 h.

Each sub-line showed variable anchorage-dependent growth as shown in Figure 2. KDM/Ud2 showed the most rapid growth with a doubling time of 23.5 h, and KDM/JuB2 showed the slowest growth with doubling time of 31.6 h.

### Expression of growth factor and growth factor receptor

**Figure 3:**
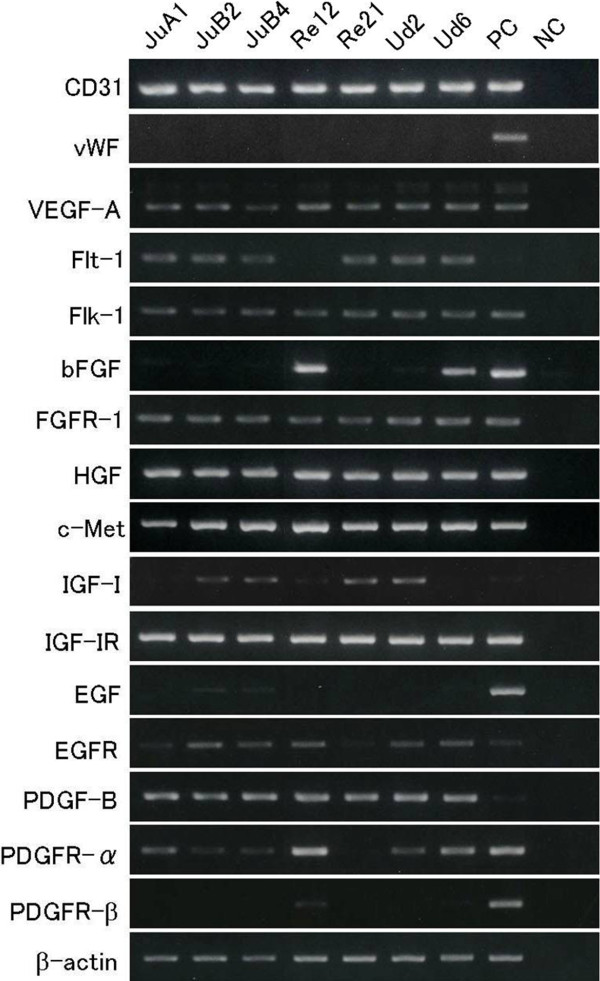
**mRNA expression of canine HSA cell lines.** RT-PCR analysis of the expression of endothelial cell-specific markers (CD31 and vWF), growth factors (VEGF-A, bFGF, HGF, IGF-I, EGF, and PDGF-B), and their receptors (Flt-1, Flk-1, FGFR-1, c-Met, IGF-IR, EGFR, PDGFR-α, and PEGFR-β). Total RNA was extracted from subconfluent cells grown in Medium 199 with 10% FBS using TRIzol reagent. β-actin was used as a loading control. mRNA extracted from canine spleen was used as PC, and water was used instead of mRNA as NC. Abbreviations: PC, positive control. NC, negative control.

The expression levels of mRNA for growth factors and their receptors were different among the cell lines as measured by RT-PCR (Figure 3). mRNAs for CD31, VEGF-A, HGF, PDGF-B, Flt-1, Flk-1, FGFR-1, c-Met and IGF-IR were detected in all cell lines, mRNA for bFGF was detected in only 2 cell lines, and no mRNA for von Willebrand factor (vWF), EGF, or PDGFR-β was detected in any cell line. Since the primer sets were generated from canine-specific sequences as previously described [25], the present results suggested that all cell lines have characteristics of canine ECs.

One cell line (KDM/Re12) had a VEGF-A concentration of 201 pg/10^6^ cells for 24 h in the cell supernatantas measured by ELISA, but bFGF was not found in the supernatant of any cell line. Immunocytochemical investigations for VEGF-A and bFGF revealed weak to moderate expression of these proteins observed in the cytoplasm of the cell lines (Figure 1B), in which the mRNA expression was found in RT-PCR.

### Effects of growth factors on cell proliferation

**Figure 4:**
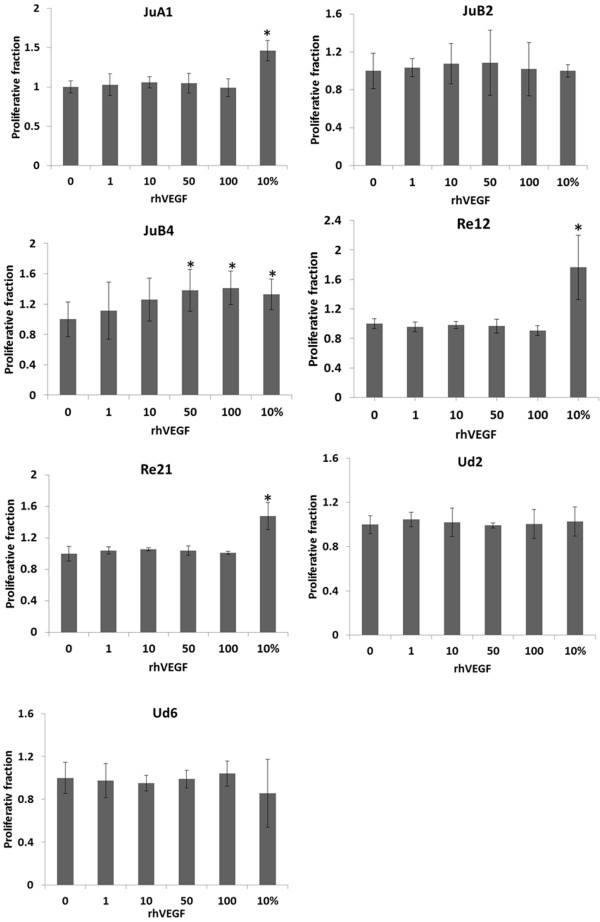
**Cell proliferation in the presence of growth factors.** Typical results of cell proliferation assays after incubation with recombinant human VEGF. Each cell line was plated at 1 × 10^3^ cells per well in 200 μL Medium 199 containing 10% FBS in 96-well plates for 24 h. After 24 h-serum starvation, 0, 1, 10, 50, or 100 ng/ml of recombinant growth factor in Medium 199 containing 1% FBS was added to the cells, or the medium was changed to Medium 199 containing 10% FBS for 72 h. The relative viable cell number was assessed by the WST-1 assay. *; p < 0.05 compared with cells that were not exposed to growth factor.

After 24 h of serum starvation, canine HSA cell lines showed differential response to growth factors, including recombinant human VEGF, bFGF, IGF-I, HGF, EGF, and PDGF-BB, recombinant canine VEGF and HGF, and to FBS as assessed by the WST-1 assay. All the cell lines could proliferate even in serum-starved condition. In KDM/JuB4, which expressed mRNA for all receptors except PDGFR-β, cell proliferation was stimulated by all growth factors except IGF-I and PDGF-BB in a dose-dependent manner, and by FBS. In KDM/JuA1, KDM/Re12, and KDM/Re21, cell proliferation was stimulated only by FBS and not by any growth factors even though these cell lines expressed mRNA for their receptors. Cell proliferation of KDM/JuB2, KDM/Ud2 and KDM/Ud6 was not stimulated by any of the growth factors or by FBS. Similar results were obtained from triplicate experiments. In CnAOECs, cell proliferation was stimulated by all growth factors except PDGF-BB and by FBS (data not shown). Figure 4 shows the typical results of cell proliferation after incubation with growth factors.

### Effects of serum stimulation on the MAPK/Erk and AKT/mTOR pathways

**Figure 5:**
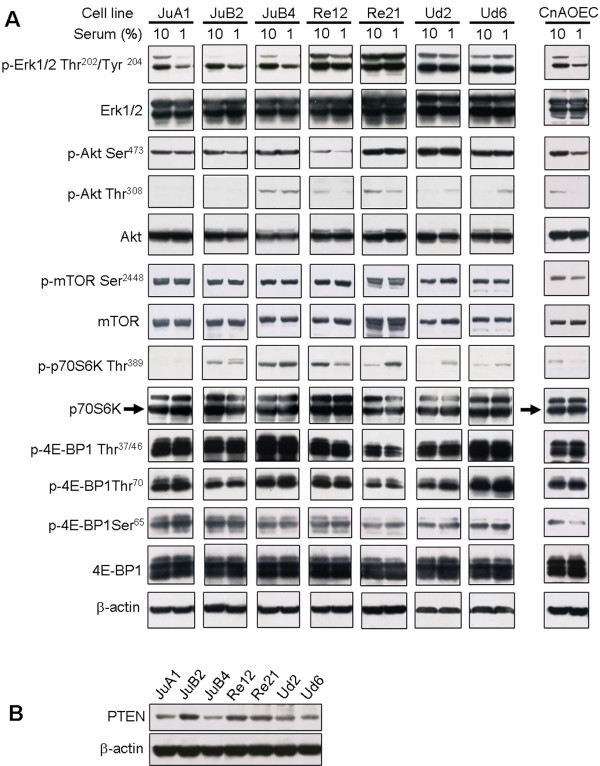
**Effects of serum stimulation on the MAPK/Erk and AKT/mTOR pathways.** (**A**) Western blot analysis for the expression of Erk, Akt, mTOR, p70S6K, and 4E-BP1. Canine HSA cell lines were grown in Medium 199 with 10% FBS. After 24 h-serum starvation, the medium was replaced with Medium 199 containing 1% FBS or Medium 199 containing 10% FBS for 30 min. The total protein was then extracted using RIPA Lysis Buffer, and equal amounts of protein (10 μg) were subjected to western blot analysis. β-actin was used as a loading control. (**B**) Western blot analysis for the expression of PTEN. The total protein was extracted from subconfluent cells grown in Medium 199 with 10% FBS using RIPA Lysis Buffer, and equal amounts of protein (10 μg) were subjected to western blot analysis. β-actin was used as a loading control.

Because cell proliferation was stimulated by FBS in 4 cell lines, we further investigated the effect of FBS on the MAPK/Erk and Akt/mTOR pathways, which are major signal transduction pathways associated with cell proliferation [4]. Western blot analysis revealed that p-p44/42 Erk1/2 Thr^202^/Tyr^204^ levels were low in serum-starved condition and increased in the presence of serum in the KDM/JuA1, KDM/JuB2, KDM/JuB4, and KDM/Re12 cell lines and a similar increase in p-p44/42 Erk1/2 Thr^202^/Tyr^204^ was observed in CnAOECs (Figure 5A). Phosphorylation levels of Akt at Ser^473^ in any cell line except KDM/Re12 were high in serum-starved condition, and FBS stimulation had no effect on its levels. Similarly, phosphorylation levels of mTORC1 at Ser^2448^ and 4E-BP1 at all residues were high in unstimulated cells and unchanged by serum stimulation in any of the cell lines. In CnAOECs, phosphorylation levels of these proteins were low in serum-starved condition, and FBS stimulation increased phosphorylation of Akt at Ser^473^, mTORC1 at Ser^2448^, and 4E-BP1 at Ser^65^ but not at Thr^37/46^ or Thr^70^. These data suggest that the phosphorylation of Akt at Ser^473^, mTORC1 at Ser^2448^, and 4E-BP1 at Ser^65^ was constitutively activated in the absence of FBS in six cell lines. The levels of p-Akt at Thr^308^ and p-p70S6K at Thr^389^ were increased by serum stimulation in KDM/Re12 cells in a manner similar to those of normal canine ECs. Conversely, FBS stimulation decreased phosphorylation of these residues in KDM/Ud2 and KDM/Ud6 cells. In addition, phosphorylation of these two sites was not affected by serum in the KDM/JuB4 cells and was not detected in KDM/JuA1 cells. The present findings suggest that the phosphorylation of p70S6K at position Thr^389^ may be related to that of Akt at Thr^308^.

Deletion or mutation of PTEN is reported in some types of tumors, including vascular tumors [9,10,29], which causes constitutive activation of the PI3K/Akt pathway. PTEN protein was detected in all cell lines. The expression levels of PTEN in the KDM/JuA1 and KDM/JuB4 cells were lower than those in other cell lines and were not related to the phosphorylation levels of Akt (Figure 5B).

### Tumor formation in nude mice

**Figure 6:**
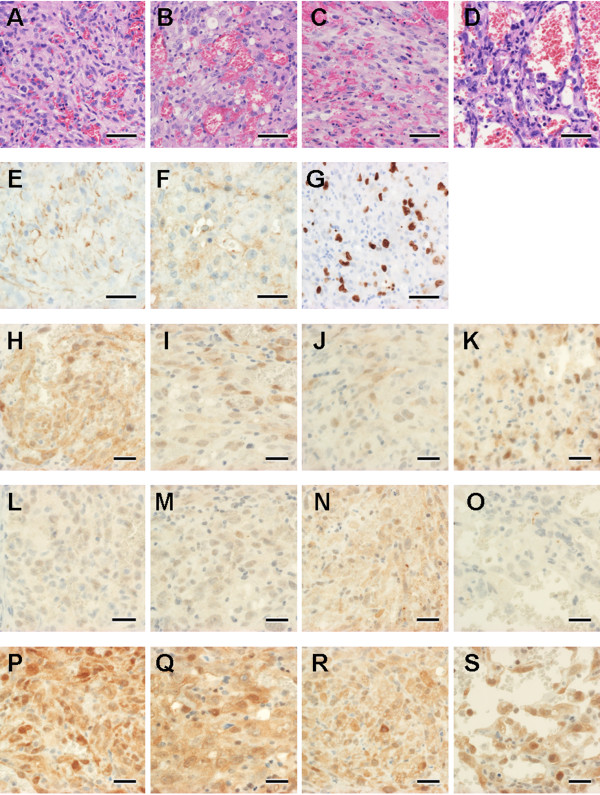
**Histology and immunohistochemical staining for EC markers and Akt/4E-BP1 in tumors formed from cell injections.** Tumors formed after injection of 1 × 10^6^ cells in the right and left dorsal area of the trunk of 3-week-old male KSN/Slc mice. (**A**-**D**) Histological features of formed tumors (A: JuA1, B: JuB2, C: JuB4, D: Re21). Hematoxylin and eosin staining; bars = 50 μm. The neoplastic cells had spindle to polygonal-shaped cytoplasm with oval nuclei, forming some areas of vascular clefts of channels. (E-S) Immunohistochemical results of CD31, vWF, Ki-67, p-Akt Ser^473^, p-Akt Thr^308^, and p-4E-BP1 Thr^37/46^ in the formed tumors. (**E**) The membrane of the tumor cells show positive staining with CD31 (JuA1). (**F**) The cytoplasm of the tumor cells show positive staining with vWF (Re21). (**G**) The positive staining of Ki-67 MIB-1 clone in the nuclei of tumor cells indicates that the tumor is not derived from the mice (JuB2). (**H**-**K**) All HSA tumors that developed showed moderate (I: JuB2, J: JuB4, and K: Re21) to strong (H: JuA1) expression for p-Akt Ser^473^ in the cytoplasm and nuclei. (**L**-**O**) HSA tumors that developed showed moderate (N: JuB4) to weak expression (L: JuA1 and M: JuB2), and one cell line (O: Re21) showed no expression of p-Akt Thr^308^. (**P**-**S**) All HSA tumors showed strong cytoplasmic and nuclear expression of p-4E-BP1 Thr^37/46^ (P: JuA1, Q: JuB2, R: JuB4, and S: Re21). Immunohistochemical staining; bars = 50 μm (G) and 25 μm (**E**, **F**, **H**-**S**).

After subcutaneous injections of cells from the various cell lines into KSN/Slc mice, tumor masses were formed in all the nude mice that had been injected with KDM/JuA1 or KDM/Re21 cells, and in 2 and 1 nude mice that had been injected with KDM/JuB2 and KDM/JuB4 cells, respectively (Table 2). No tumor masses were formed with injection of KDM/Re12, KDM/Ud2, or KDM/Ud6 cells. No metastasis was observed after injection with any of the cell lines during experimental periods and, histologically, all the tumor masses that developed showed vascular tissue-like structures (Figure 6A–D). The tumor tissues formed by KDM/Re21 injection showed incomplete larger vascular-like structures (Figure 6D) than those formed form other cell lines. Because the formed tumors contained many types of cells, such as inflammatory cells, in which similar signaling pathways may be activated as those in tumor cells, it was difficult to evaluate the protein expression of tumor cells alone by western blot analysis. Therefore, we performed immunohistochemistry to examine the localization of protein expression. All tumors showed positive reactivity for CD31 (Figure 6E) and vWF (Figure 6F), and positive reactivity for Ki-67 antigen of MIB-1 clone (Figure 6G) was observed in the nuclei of the tumor cells, but no positive reactions were observed in the surrounding murine tissues such as the epidermal basal cells. Because murine tissues do not react with the antibody against Ki-67 antigen of MIB-1 clone [30], the positive reactivity for both Ki-67 antigen of MIB-1 clone and EC markers in the tumor cells provided evidence that the tumor masses that formed in the nude mice were not derived from the original tissues in the mice and were HSAs induced by cell injections.

**Table 2:** **Tumor growth after subcutaneous injection of 1 × 10**^**6**^**cells of each canine HSA cell line**

	**Growth (No. of animals)**	**Growth (No. of tumors)**	**Mean volume (mm**^**3**^**)**
JuA1	5	8	102.0
JuB2	2	3	88.6
JuB4	1	2	8.75
Re21	5	9	58.8

Tumor volume is calculated: (width)^2^ × length/2 mm^3^.

All tumors that formed were examined further for expression of the Akt/4E-BP1 pathway. Moderate to intense degrees of phosphorylation of Akt at Ser^473^was observed in both the nuclei and cytoplasm in all tumors (Figure 6H–K). On the other hand, weak to moderate phosphorylation of Akt at Thr^308^ was observed in both the nuclei and cytoplasm (Figure 6L–N), and this phosphorylation was not detected in tumors formed from Re21 injections (Figure 6O). 4E-BP1 at Thr^37/46^was highly phosphorylated in both the nuclei and cytoplasm in all tumors (Figure 6P–S).

## Discussion

We established 7 canine HSA cell lines from nude mice-xenograft canine HSAs [25]. Although all original canine HSA xenograft tumors expressed mRNA for bFGF [25], some sub-lines derived from the same xenograft tumor lacked expression of bFGF. The differences in expression between xenograft tumors and subsequently derived sub-lines suggested that each xenograft tumor might contain a variety of tumor cells with different phenotypes. Each cell line had characteristics of ECs, which was confirmed by expression of CD31 mRNA and incorporation of DiI-Ac-LDL. However, vWF mRNA was not detected in any of the cell lines. The loss of vWF has also been reported in human angiosarcomas and canine HSA cell lines [22,23] and occurs in undifferentiated malignant ECs [31]. Therefore, the expression of vWF is of limited value for identifying malignant ECs [22], and CD31 is the most reliable EC marker [32,33]. Unlike the expression levels in the cultured cell lines, expression of vWF and CD31 was observed in the tumors that formed after cell injections. vWF is produced by ECs and megakaryocytes, and adhere to collagen in the subendothelium [34]. Tumors that formed after cell injection contained not only tumor cells but diverse cells, including red blood cells, inflammatory cells, and stromal cells. These cellular constituents may account for the differences in vWF expression observed between cultured cell lines and the resulting tumors after injection with these cells, but the exact cause of the differences remains unclear.

The established canine HSA cell lines expressed differing levels of mRNA for a variety of growth factors and their receptors. Although receptors were expressed in most of the cell lines, cell proliferation was stimulated only by the associated growth factors in the case of KDM/JuB4, in which proliferation was also stimulated by serum. Stimulated proliferation of 3 cell lines was observed in the presence of serum alone. A previous study with a canine HSA cell line showed that proliferation was stimulated by serum and the same growth factors that we used except for human VEGF and PDGF-BB [24]. The previous study had a limitation, in that it analyzed only a single cell line. Because the present cell lines expressed both growth factors and their receptors, the lack of response to the growth factors may be the result of saturation of the receptors by growth factors in an autocrine or paracrine manner. Our findings suggest that serum may be a potent stimulator of cell proliferation in diverse types of canine HSA cells. In the serum, interleukins (ILs) such as IL-1α and IL-8 may be the primary stimulator since they are known to stimulate cell growth in canine HSAs as well as in normal ECs [35,36]. However, a limitation of this study is that we could not evaluate the protein expression of receptors. Another possibility is that the lack of protein expression of the receptors may lead to unstimulated proliferation regardless of the mRNA expression.

In the present study, VEGF was detected in culture supernatant only in one cell line, even though mRNA and protein for VEGF was detected in all cell lines, and bFGF was not detected in the supernatant of any cell lines, including two cell lines that expressed mRNA and protein for bFGF. VEGF is known to regulate normal angiogenesis [37,38] and is overexpressed in vascular tumors of both humans and dogs [2,3]. In the previously reported canine HSA cell lines, VEGF [23,24] and a small amount of bFGF [24] were detected using the same ELISA kit as that used in the present study. However, another study found that even though VEGF was present at high levels in the cytoplasm of activated ECs, it could not be detected in culture supernatant due to low levels of extracellular release [36]. Because VEGF and bFGF mRNA and protein were expressed in the present cell lines but not in the supernatant, these growth factors are most likely to be contained only in the cytoplasm and were not released into the cell supernatant. It is also unknown whether these growth factors are released into the extracellular matrix in spontaneously occurring canine HSAs, in which both VEGF and bFGF are overexpressed [3].

The phosphorylation of Akt at Ser^473^ was not affected by FBS stimulation in all cell lines except KDM/Re12. In addition, the phosphorylation of mTORC1 at Ser^2448^ and 4E-BP1 at all residues was unchanged in all cell lines. In normal canine ECs, the phosphorylation of Akt at Ser^473^, mTORC1 at Ser^2448^, and 4E-BP1 at Ser^65^ was increased in the presence of FBS, but not phosphorylation of 4E-BP1 at Thr^37/46^ or Thr^70^. 4E-BP1 is known to be sequentially phosphorylated on three residues: phosphorylation of Thr^37/46^ is followed by Thr^70^ and then Ser^65^[15]. The phosphorylation of Thr^37/46^ is relatively unaffected by serum [39], whereas phosphorylation of Thr^70^ and Ser^65^ are stimulated by serum [15]. However, a recent study indicated that different cell types as well as different stimuli lead to different 4E-BP1 phosphorylation [40]. Furthermore, Ser^65^ of 4E-BP1 is an essential site for the control of translation initiation by release of 4E-BP1 from eIF4E [15]. Our results suggest that phosphorylation of 4E-BP1 at Ser^65^ was the only site that was regulated in a serum-dependent manner in normal canine ECs, rather than Thr^37/46^ and Thr^70^. This indicates that Ser^65^ of 4E-BP1, Ser^473^ of Akt, and Ser^2448^ of mTORC1 were constitutively activated in the present cell lines. mTORC1 and mTORC2 are located both upstream and downstream of Akt, and Ser^473^ of Akt is directly phosphorylated by mTORC2 [19], whereas mTORC1 at Ser^2448^ is phosphorylated by Akt [14]. The present findings suggest that the mTORC2/Akt/4E-BP1 pathway was constitutively activated in a serum-independent manner, and was considered to be deregulated in the present cell lines compared with that in normal ECs. Consistent with the present results, constitutive phosphorylation of both Akt at Ser^473^ and 4E-BP1 is reported in lymphomas [29] and acute myeloid leukemia [41]. Since these constitutively activated pathways are highly sensitive to molecular targeted therapies [5], the mTORC2/Akt/4E-BP1 pathway may be a novel target for treatment of canine HSAs. However, there is still possibility that mTORC1 and 4E-BP1 are phosphorylated independently of mTORC2, because mTORC1 was unaffected by serum regardless of increased phosphorylation of Akt at Ser^473^ in KDM/Re12. Another possibility is that phosphorylation of 4E-BP1 may not be caused by Akt nor mTORC1 because 4E-BP1 is known to be phosphorylated by p44/42 Erk1/2 [42]. This is most likely to occur in KDM/Ud2 and KDM/Ud6 because the phosphorylation of Erk1/2 was unchanged in the presence of FBS.

Although 4E-BP1 was constitutively activated independent of FBS, cell proliferation was stimulated by serum in 4 cell lines. This stimulation seemed to be related to increased phosphorylation of p44/42 Erk1/2 Thr^202^/Tyr^204^, similar to that of normal canine ECs. The MAPK/Erk pathway regulates cell proliferation differently from the PI3K/Akt pathway [4,5] and is not activated in human angiosarcomas [7]. In contrast, the mTORC2/Akt/4E-BP1 pathway may regulate serum-independent cell proliferation because HSA cells could grow in serum-starved conditions. Another possibility is that constitutive mTORC2/Akt/4E-BP1 activation may lead to other effects besides cell proliferation since mTOR also regulates the cell cycle and anti-apoptosis [12,13]. In KDM/Ud2 and KDM/Ud6, both the MAPK/Erk and mTORC2/Akt/4E-BP1 pathways were constitutively phosphorylated, and FBS stimulation failed to stimulate cell proliferation. RTKs are well-known activators of the MAPK/Erk and Akt/mTOR pathways, and mutations of RTKs in cancer lead to constitutive activation of these pathways [4,5]. Therefore, the present constitutive activation of these two pathways may be result from aberrant activation of RTKs.

As opposed to phosphorylation of Akt at Ser^473^, the phosphorylation of Akt at Thr^308^ was affected by FBS stimulation and seemed to be correlated with the phosphorylation of p70S6K. Akt is usually phosphorylated at Thr^308^ by 3- phosphoinositide-dependent kinase, whereas Ser^473^ is phosphorylated by mTORC2 [20]. Although both p70S6K and 4E-BP1 are located downstream of mTORC1 [13], recent studies have indicated that these 2 proteins are regulated by distinct signaling pathways in some types of cells. In normal ECs, p70S6K is regulated by mTORC1, and 4E-BP1 is regulated by Akt independently of the mTORC1 pathway [40]. The mTORC1-independent regulation of 4E-BP1 has been also demonstrated in hematopoietic malignancies [29,41]. Taken together, the phosphorylation of p70S6K and 4E-BP1 in the present cell lines was probably regulated by 2 different signaling pathways.

Deletion or mutation of PTEN is known to cause constitutive activation of the PI3K/Akt pathway in some types of tumors, including vascular tumors [9,10,29]. Deletion or point mutations have been reported in the C-terminal domain of PTEN in canine HSA cell lines [9]. The antibody used in the present study also recognizes the C-terminal domain of PTEN. We found no evidence for deletion of PTEN in the present cell lines, despite constitutive phosphorylation of Akt at Ser^473^. It is known that constitutive activation of Akt is not always associated with the deletion or mutation of PTEN [9,43], and other growth factors and signaling pathways are suggested to regulate the constitutive activation of this pathway [43]. However, we were unable to test for mutations of PTEN, and there is a possibility that a mutation in PTEN was associated with the constitutive activation of Akt.

After cell injections into nude mice, HSA tumors developed from 4 cell lines. In these mice with developed tumors, no metastatic lesion was observed, similar to that of original canine HSA xenograft models [25]. Similarly, metastatic tumor was not detected after subcutaneous injection of the human angiosarcoma cell line in nude mice despite tumorigenicity on the skin. Canine HSAs [1] as well as human angiosarcomas [2] have high metastatic biology that leads to poor prognosis; however, the established cell lines did not show these characteristics. Another study of a canine HSA cell line indicated that intravenous injection formed metastatic lesion in the lungs of SCID mice [35]. The differences in the results of metastasis may depend on the route of cell injection or immunity of mice. Another possibility is that the metastatic property may be lost during passages of xenograft tumor or cell culture. However, immunohistochemical analysis in the present study revealed that the developed tumors after cell injection had high levels of phosphorylation of Akt at Ser^473^ and 4E-BP1 at Thr^37/46^ similar to that of the original cell lines. These *in vivo* models would be useful tools for evaluating the anti-tumor effect of inhibitors targeting the mTORC2/4E-BP1 pathway. Drugs targeting both mTORC2 and mTORC1 have been studied in acute myeloid leukemia and have shown marked anti-tumor effects [44]. Because both mTORC1 and mTORC2 are activated during angiogenesis [45], mTORC1/mTORC2 inhibition may have a potent effect in HSA tumors through inhibition of not only tumor cell proliferation but also angiogenesis.

## Conclusions

We have established 7 canine HSA cell lines from 3 xenograft canine HSAs. These cell lines showed diverse morphologies and mRNA expression levels for VEGF-A, bFGF, HGF, IGF-I, EGF, and PDGF-B and their receptors. Cell proliferation was stimulated by these growth factors and FBS in one cell line, was stimulated by FBS alone in 3 cell lines, and was not stimulated by either growth factors or FBS in the remaining 3 cell lines. Phosphorylation of p44/42 Erk1/2 was increased in the presence of FBS in 4 cell lines and seemed to be related to serum-dependent proliferation. In contrast, phosphorylation of Akt at Ser^473^, mTORC1 at Ser^2448^, and 4E-BP1 at Ser^65^ was not altered by FBS stimulation in 6 cell lines, suggesting that the mTORC2/Akt/4EBP1 pathway was constitutively activated in the present cell lines. After cell injection into nude mice, canine HSA tumors were formed in 4 cell lines. These tumors showed similar expression levels for phosphorylated Akt and 4E-BP1 as the original cell lines. Therefore, the present cell lines are useful models to investigate the role of the mTORC2/Akt/4E-BP1 pathway in canine HSA in both *in vitro* and *in vivo *systems.

## Competing interests

The authors declare that they have no competing interests.

## Authors’ contributions

AK was involved in study design, establishment of cell lines, and histopathology. HS and SAA conceived the aims of study and participated in its design and coordination. AH and TY participated in study coordination. All authors have read and approved the final manuscript.
